# The impact of arthritis on the physical function of a rural Maya-Yucateco community and factors associated with its prevalence: a cross sectional, community-based study

**DOI:** 10.1007/s10067-015-3084-x

**Published:** 2015-10-07

**Authors:** Adalberto Loyola-Sanchez, Julie Richardson, Ingris Pelaez-Ballestas, José Alvarez-Nemegyei, John N. Lavis, Michael G. Wilson, Seanne Wilkins

**Affiliations:** 1 School of Rehabilitation Science, McMaster University, Hamilton, ON Canada; 2 Rheumatology Department, Hospital General de México, Ciudad de México, Mexico; 3 Research Unit, Hospital Regional de Alta Especialidad de la Península de Yucatán, Mérida, Mexico; 4 Centre for Health Policy Analysis, McMaster University, Hamilton, ON Canada; 5 McMaster Health Forum, McMaster University, Hamilton, ON Canada; 6 Department of Clinical Epidemiology and Biostatistics, McMaster University, Hamilton, ON Canada; 7 Department of Political Science, McMaster University, Hamilton, ON Canada; 8 Department of Global Health and Population, Harvard School of Public Health, Boston, MA USA; 9 Institute for Applied Health Sciences, Room 403, 1400 Main St. W., Hamilton, ON L8S 1C7 USA

**Keywords:** Arthritis, Disability evaluation, Indians North American, Mexico, Observational study

## Abstract

This study aims to evaluate the impact of arthritis on the physical function of people living in a Maya-Yucateco rural community and to assess the association of known modifiable risk factors with the prevalence of overall arthritis and its main types (osteoarthritis and rheumatoid arthritis). Using a cross-sectional, community-based census design, data collected from the adult population (≥18 years) of the Municipality of Chankom, Yucatán, México, were analyzed (*n* = 1523). Participants’ physical function was assessed using a culturized version of the health assessment questionnaire disability index. Social, physical, and behavioral factors linked to overall arthritis, osteoarthritis, and rheumatoid arthritis, were assessed through the “Community-Oriented-Program-for-the-Control-of-Rheumatic-Diseases [COPCORD]” questionnaire. A physiatrist and a rheumatologist confirmed all osteoarthritis and rheumatoid arthritis cases using the American College of Rheumatology criteria. Arthritis was confirmed in 169 cases (22 %, 95 % confidence interval (CI) 19–25) of those assessed for musculoskeletal symptoms (*n* = 779): osteoarthritis = 144, rheumatoid arthritis = 17, and non-specific arthritis = 8. Arthritis was associated with a higher prevalence of disability after controlling for age, gender, and number of comorbidities (odds ratio = 4.0, 95 % CI 3.0–6.0). Higher level of wealth was associated with lower arthritis prevalence (odds ratio = 0.9, 95% CI 0.8–0.9). Higher body mass index was associated with higher hip and/or knee osteoarthritis prevalence (odds ratio = 1.1, 95 % CI 1.03–1.1). Arthritis is highly associated with disability in the Mayan people living in Chankom. The prevalence of arthritis in Chankom is associated with social factors, such as people’s level of wealth, while the prevalence of low-extremity osteoarthritis is associated with people’s body mass index.

## Introduction

Disability is a public health priority [1]. The level of disability is inversely associated with socioeconomic position [2]. Musculoskeletal (MSK) diseases are the most common cause of disability worldwide [3] and their disabling effects are more severe in developing countries [4].

Arthritis is a common term used to group different chronic MSK diseases of the joints, mainly osteoarthritis (OA) and rheumatoid arthritis (RA) [5, 6]. Arthritis is considered a leading cause of disability [5]. One out of nine persons with arthritis experience disease-related limitations in fulfilling their life roles [7].

The level of disability associated with arthritis is higher for people living in low socioeconomic conditions [7, 8]. For example, the functional status of people living with knee and/or hip OA in middle- and low-income countries is lower than those living in high-income countries [9]. Consequently, the negative effects of arthritis are likely to be associated with social determinants linked with increased disability within low-socioeconomic areas in developing countries.

Considered together, OA and RA are the leading MSK diseases in the Mexican Southern state of Yucatán [10]. This state includes several indigenous rural communities from the Maya-Yucateco culture. Mexican indigenous communities experience low socioeconomic living conditions and limited access to appropriate health care services [11]. As a result, these communities are more vulnerable to the disabling effects of arthritis, underscoring the need to develop rehabilitation interventions aimed at preventing and decreasing these effects.

Developing rehabilitation interventions for arthritis requires an understanding of its impact on physical function and identifying modifiable factors associated with the manifestation of this condition. This will help in designing actions to decrease the prevalence and prevent the disabling effects of arthritis at the community level. This study was part of a project designed to develop a community-based rehabilitation program to ameliorate the disabling effects provoked by MSK diseases in a rural, lower-socioeconomic, and underserved Maya-Yucateco community in Yucatán [12].

The objectives of this study were to evaluate the impact of arthritis on the physical function of people living in the Mayan municipality of Chankom, Yucatán, México, and to evaluate the association between modifiable factors and the prevalence of overall arthritis and its main types (OA and RA) in this community.

## Materials and methods

### Study design

**Fig. 1:**
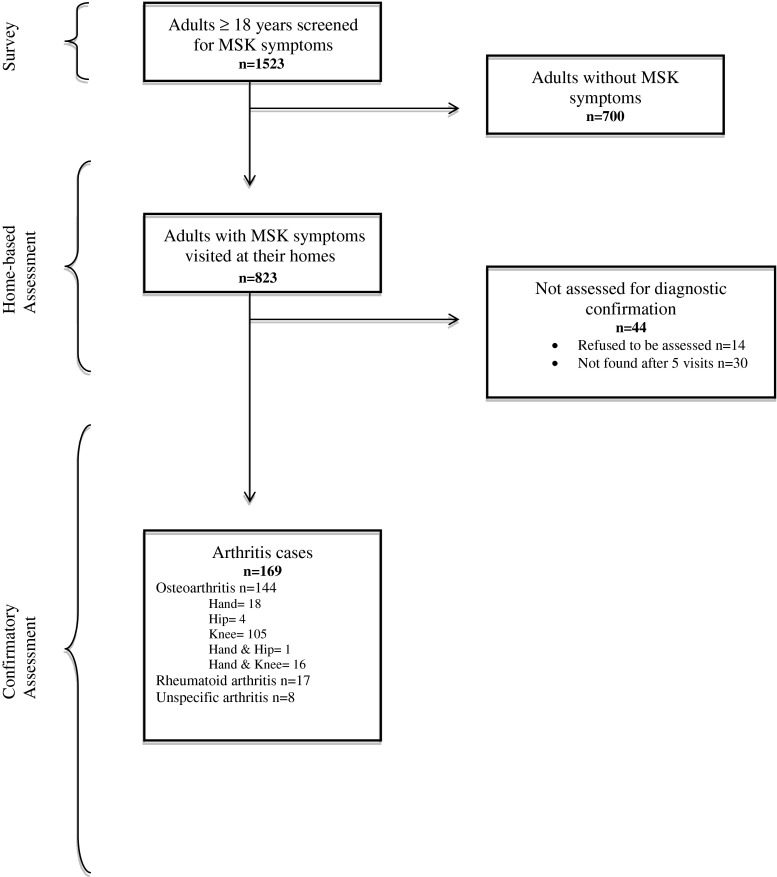
Study stages and participants’ flowchart

This was an observational, cross-sectional, community-based study undertaken in three stages: (a) survey, (b) home-based assessment, and (c) confirmatory assessment (see Fig. 1). These stages were based on the Community Oriented Program for the Control of Rheumatic Diseases (COPCORD) phase one methodology, as described elsewhere [13] (www.copcord.org). The survey stage consisted of a census conducted in the adult population (≥18 years) of the municipality of Chankom, Yucatán. Trained local personnel applied a cross-culturally validated questionnaire [14] designed to detect MSK symptoms and quantify relevant clinical and socioeconomic factors. Two family physicians, trained in rheumatologic evaluation and assisted by local translators, assessed all people who reported MSK symptoms in their homes (home-based assessment stage) within the same week in which the surveys had been applied. During the confirmatory assessment stage, a physiatrist and a rheumatologist evaluated all possible OA or RA cases, respectively, within 1 month of the initial contact and with the help of local Mayan translators.

This study was approved by the Hamilton Health Sciences/McMaster University Research Ethics Board (12–544), the Ethics Committee of the Faculty of Medicine, Universidad Anáhuac-Mayab, and the Ethics Committee of the “Hospital General de México, Dr. Eduardo Liceaga” (DI/11/4044B/3/123). All participants signed an informed consent before participating in the study.

### Setting

This study was conducted in the rural municipality of Chankom, which is located at the Southeast of Yucatán, Mexico, and comprised of 11 small rural villages inhabited entirely by people of the Maya-Yucateco ethnicity. Chankom is situated on a flat rocky land of tropical forest, 27 m above the sea level with a predominantly warm humid climate. The municipality of Chankom has a population of 4464 habitants of whom 80 % are considered to be living in poverty [15]. The three stages were conducted between June and December 2012. The confirmatory assessments were performed at various locations, including people’s homes, villages’ public facilities, and a municipal evaluation center. People identified as having possible OA received a radiographic evaluation at the nearest public hospital.

### Participants

Osteoarthritis diagnosis in participants were confirmed by the physiatrist following the American College of Rheumatology (ACR) clinical criteria for hand OA [16] and radiological and clinical criteria for hip and knee OA [17, 18]. A rheumatologist certified by the Mexican Board of Rheumatology and with ≥15 years of clinical practice evaluated all participants detected with inflammatory arthritis and confirmed all cases of RA. Cases with clear signs of arthritis but which did not fulfill OA or RA criteria were classified as “unspecific arthritis.”

### Measurements

#### Physical function

Physical function was evaluated through the health assessment questionnaire disability index (HAQ-DI) applied during the survey stage. The HAQ-DI has shown good psychometric properties when used in OA [19] and RA [20] populations. This instrument was cross-culturally translated, adapted, and validated for use in the Maya-Yucateco population [14] and demonstrated good test-retest reliability (ICC = 0.69) when applied to a sample of 30 individuals living with MSK diseases in Chankom [21]. The HAQ-DI was scored following standard procedures [22] and then transformed to a dichotomous variable to indicate the presence or absence of disability using a cutoff point score of 0.25, as reported in a population-based study [23].

#### Modifiable risk factors

Arthritis prevalence has been associated with several modifiable social, physical, and behavioral factors [24, 25]. On the one hand, OA prevalence has been associated with socioeconomic factors (i.e., gender, race, and nutrition) that provoke joint vulnerability, and with factors that increase joint loading, such as body mass index (BMI) and the execution of repetitive movements, which augments the mechanical stress within the joints [26]. On the other hand, RA prevalence has been associated with low level of wealth, high BMI, and smoking [25, 27].

The participants’ level of wealth, or property that can be sold and converted to cash for the benefit of the owner, was assessed as a socioeconomic status variable during the survey stage. Property owned by participants was registered, selected, and classified by local staff according to the type of properties that better differentiate between people’s level of wealth in the community. Property related to entertainment, electro-domestic appliances, communication, and transportation were organized into a hierarchical format and combined to derive a “level of wealth” variable ranging from 0 “no properties owned” to 14 “ownership of the highest-valued properties.”

Body mass index was registered as the Quetelet’s index (weight/height^2^). Weight and height were measured during the survey stage following Lohman’s technique [28]. Weight was measured with a portable digital scale (Tanita Model 804), and height was measured with a portable ultrasonic digital stadiometer (ADE, Ultraschall/Messstab/MZ10020, Germany).

Finally, the following behavioral variables were assessed during the survey: self-reported smoking status, as a yes/no question, and the regular performance of repetitive movements, by asking participants to define their lifetime’s principal occupation and whether this occupation involved frequent repetitive movements such as: jolting hands, lifting or pushing ≥20 kg, climbing, standing, kneeling for longer than 30 min, and constant shifting from sit-to-stand positions. Two variables were then created to reflect the cumulative mechanical stress within the joints, one for static stress formed by standing and kneeling longer than 30 min (0 to 2) and one for dynamic stress formed by jolting hands, lifting, and pushing ≥20 kg, climbing stairs or slopes, and constant shifting from sit-to-stand positions (0–5).

#### Confounders

Factors known to be associated with physical function such as age, gender, and number of comorbidities were evaluated at the survey stage through self-report. These factors were considered to be confounders for determining the association between arthritis and disability after no interaction effects were found between these and the association of interest [29]. The number of comorbidities was determined by adding the number of reported diseases by participant from a list including: diabetes, hypertension, cardiovascular disease, dyslipidemia, gastritis, anxiety and depression, resulting in a continuous variable ranging from 0 to 7.

### Statistical analyses

Descriptive statistics were generated through the calculation of means and frequencies. *T* tests adjusting for unequal variances (Satterthewaite’s approximation) and chi^2^ tests were used to compare means and frequencies between those participants assessed or not for diagnostic confirmation and between participants with and without arthritis. A logistic regression model was used to evaluate the relationship between the presence of overall arthritis (independent variable) and the presence of disability (dependent variable), adjusting for previously described confounders.

Modifiable risk factor analyses were conducted separately for overall arthritis, OA, and RA using logistic regression models considering the presence of disease as the dependent variable and all the factors evaluated as the independent variables. The OA analysis was adjusted for age and gender. Gender-based subgroup analyses were conducted for hand and lower extremity (hip and/or knee) OA, adjusting by age. Penalized maximum likelihood estimation (Firth’s method) was used to estimate logistic regression parameters and profile penalized likelihood confidence intervals in the RA, hand and lower extremity OA analyses, accounting for the observed phenomenon of complete separation in the data [30].

All regression models were constructed following a complete-case analysis strategy. Assumptions and the models’ goodness of fit were confirmed using likelihood ratio tests, hat^2^ tests, Hosmer and Lemenshow tests, and the area under the curve. Hypothesis testing was deemed statistically significant at *α* = 0.05. Two statistical analysis packages (STATA 12.1. and R 3.1.1) were used.

## Results

Fifteen hundred and twenty three adults answered the questionnaire during the survey stage and 823 (54 %) reported MSK symptoms. Forty-four participants (5 %) who reported MSK symptoms could not be seen by a specialist during the confirmatory assessment stage because either they refused or were not found at their homes after five visits and were excluded from the analysis. Proportionally, more men (70 vs. 39 %, Chi^2^_[1]_ = 17.78, *p* < 0.0001) and more smokers (21 vs. 9 %, Chi^2^_[1]_ = 5.95, *p* = 0.01) were not assessed for diagnostic confirmation. Arthritis was confirmed in 169 cases (22 %): 144 with OA, 17 with RA, and 8 with unspecific arthritis (Fig. 1).

Table 1 shows the general characteristics of the population. The mean age of the whole population was 45 years, while for the group of OA and RA, the means were 63 and 55 years, respectively. In total, 61 % of the population was female, whereas 74 % of the hand OA and 76 % of the RA groups were women. The OA population reported twice the average number of comorbidities than the rest of the population. Mean BMI was higher in the arthritis group than in the rest of the population. The smoking prevalence in the whole population was 9 %, while 0 % of the RA population reported this behavior. On average, level of wealth was significantly higher in the non-arthritis group (6 vs. 5, *T* = 3, *n* = 1478, *p* = 0.002).

**Table 1:** Participants’ general characteristics

	Total^a^ (*n* = 1479)	Arthritis^b^		
		Hand OA^c^ (*n* = 35)	Hip or knee OA^c^ (*n* = 126)	RA (*n* = 17)
Mean years of age (SD)	45 (18)	63 (12)	63 (13)	55 (13)
Female (%)	904 (61)	26 (74)	68 (54)	13 (76)
Mean comorbidities (SD)	1 (1.2)	2 (1)	2 (1)	1 (0.4)
Mean BMI (SD)	28 (5) [missing: 22]	29 (5)	29 (5)	29 (4) [missing:1]
Smokers (%)	139 (9)	2 (6)	10 (8)	0 (0)
Mean level of wealth (SD)	6 (3) [missing 1]	5 (3)	5 (3)	5 (2)
Disability (%)	251 (17)	23 (63)	60 (48)	10 (59)

*SD* standard deviation, *BMI* body mass index, *missing* number of participants with missing data, *OA* osteoarthritis, *RA* rheumatoid arthritis

^a^Data from participants not assessed for diagnostic confirmation are not included

^b^Data from participants diagnosed with non-specific arthritis are not described

^c^Cases with combined hand and hip or hand and knee OA were considered in the estimation of both the hand and the hip or knee osteoarthritis parameters

Disability was present in 165/1310 participants without arthritis (13 %) and 86/169 participants with arthritis (51 %). Disability frequencies per arthritis subgroup are shown in Table 1. Having arthritis was significantly associated with having disability after adjusting for gender, age, and number of comorbidities [prevalence odds ratio (POR) 3.8 (95 % confidence interval (CI) 2.6–5.6); Wald test 5.1, *p* < 0.0001].

Table 2 shows the frequency of participants’ performance of regular repetitive movements during their main occupations. All men with hand OA reported doing repetitive jolting-hand and sit-to-stand movements. Performance of repetitive activities such as lifting, pushing, climbing, standing, kneeling, walking, and sitting-to-standing were reported less often in the RA group than in the non-arthritis group.

**Table 2:** Frequency of regular repetitive movements performance during principal occupation

	Arthritis					No arthritis^b^
	Hand OA^a^		Hip or knee OA^a^		RA	
	Women (*n* = 26)	Men (*n* = 9)	Women (*n* = 68)	Men (*n* = 58)	(*n* = 17)	(*n* = 1310)
Jolting hands (%)	21 (81)	9 (100)	54 (79)	47 (81)	14 (82)	981 (76) [missing 11]
Lifting 20 kg (%)	10 (38)	5 (56)	30 (44)	46 (79)	4 (24)	777 (60) [missing 11]
Pushing 20 kg (%)	11 (42)	6 (67)	34 (50)	45 (78)	5 (29)	846 (65) [missing 12]
Climbing (%)	12 (46)	4 (44)	36 (53)	24 (41)	4 (24)	635 (49) [missing 12]
Standing >30 min (%)	19 (73)	8 (89)	60 (88)	46 (79)	5 (29)	1169 (90) [missing 12]
Kneeling >30 min (%)	14 (54)	6 (67)	42 (61)	39 (67)	7 (41)	786 (61) [missing 12]
Sit-to-stand (%)	19 (73)	9 (100)	57 (83)	50 (86)	11 (65)	1080 (83) [missing 12]

*OA* osteoarthritis, *RA* rheumatoid arthritis, *missing* number of participants with missing data

^a^Cases with combined hand and hip or hand and knee OA were considered for both hand and hip or knee OA parameters’ estimation

^b^ Includes data from participants without musculoskeletal symptoms and participants diagnosed with other than arthritis during the confirmatory stage

Prevalence odds ratios expressing the associations between modifiable risk factors and the presence of arthritis, OA, and RA are presented in Table 3. Body mass index was directly associated with higher arthritis prevalence, while levels of wealth and static cumulative mechanical joint stress were associated with lower arthritis prevalence. Only BMI was significantly associated with a higher prevalence of OA, and only static cumulative mechanical joint stress was significantly associated with a lower prevalence of RA.

**Table 3:** Prevalence odds ratios for the associations between selected risk factors and arthritis, OA and RA

Risk factors	Arthritis (95 % CI)	OA (95 % CI)^a^	RA (95 % CI)^b^
BMI	1.1 (1.03, 1.1)*	1.01 (1.06, 1.15)*	1.0 (0.9, 1.1)
Wealth	0.9 (0.8, 0.9)*	1.1 (0.9, 1.1)	0.9 (0.7, 1.1)
Static mechanical stress	0.7 (0.5, 0.9)*	0.1 (0.7, 1.3)	0.3 (0.1, 0.6)*
Dynamic mechanical stress	1.0 (0.9, 1.2)	1.1 (0.9, 1.3)	1.0 (0.7, 1.4)
Smoking	0.7 (0.4, 1.3)	0.1 (0.5, 1.9)	0.4 (0, 3.0)

*BMI* Body mass index, *OA* osteoarthritis, *RA* rheumatoid arthritis

^a^Adjusted by age and gender

^b^Firth logistic regression and profile penalized likelihood confidence intervals

*Significant at *α* = 0.05

Adjusted by age prevalence odds ratios estimated during the OA subgroup analyses are presented in Table 4. Body mass index was significantly associated with a higher prevalence of lower extremity OA in women and men. Repetitive lifting of ≥20 kg was significantly associated with a lower prevalence of hand OA in men. Finally, repetitive standing for longer than 30 min was significantly associated with a lower prevalence of lower extremity OA in men.

**Table 4:** Adjusted prevalence odds ratios for the associations between selected risk factors and hand OA and hip or knee OA in women and men

Risk factors	Hand OA (95 % CI)^a^		Hip or knee OA (95 % CI)^a^	
	Women	Men	Women	Men
Wealth	1.0 (0.9, 1.2)	0.9 (0.7, 1.3)	0.9 (0.9, 1.0)	1.0 (0.9, 1.1)
BMI	1.1 (0.9, 1.1)	1.0 (0.8, 1.2)	1.1 (1.1, 1.2)*	1.1 (1.1, 1.2)*
Smoking	0.70 (0.0, 11.4)	1.4 (0.2, 5.7)	2.4 (0.2, 16.6)	0.9 (0.4, 1.9)
Jolting hands	1.8 (0.7, 5.7)	4.9 (0.6, 609)	1.4 (0.7, 2.8)	1.3 (0.6, 2.9)
Lifting ≥20 kg	1.2 (0.3, 5.7)	0.1 (0.0, 0.8)*	1.1 (0.4, 2.7)	0.8 (0.2, 4.8)
Pushing ≥20 kg	0.9 (0.2, 3.5)	7.5 (0.4, 129)	1.0 (0.4, 2.4)	1.5 (0.3, 8.5)
Climbing	1.7 (0.6, 4.7)	1.2 (0.3, 5.4)	1.5 (0.8, 2.9)	1.0 (0.5, 2.0)
Standing >30 min	0.4 (0.1, 1.2)	1.5 (0.1, 20.2)	1.3 (0.5, 3.6)	0.3 (0.1,0.7)*
Kneeling >30 min	1.0 (0.4, 2.7)	0.6 (0.1, 3.5)	0.9 (0.5, 1.9)	1.4 (0.6, 3.2)
Sit-to-stand	0.5 (0.2, 1.4)	3.2 (0.3, 447)	0.9 (0.4, 2.0)	1.0 (0.4, 2.6)

Adjusted by age

*OA* osteoarthritis, *BMI* Body mass index, *CI* confidence interval

^a^Firth logistic regression and profile penalized likelihood confidence intervals

*Significant at α = 0.05

## Discussion

### Principal findings

Overall, the presence of arthritis is common in the municipality of Chankom, which aligns with what has been reported in other Mexican [31] and international reports [6]. The disability prevalence ratio between the arthritis and non-arthritis populations is 2.8:1, as calculated by Zhang’s method [32]. This means that people living with arthritis in this community are 2.8 times as likely to have disability as the people living without arthritis after controlling for age, gender, and number of comorbidities. Consequently, this group of chronic conditions have important disabling effects in this community, as has been observed in other populations [7].

The results from the evaluation of associations between modifiable risk factors and the overall prevalence of arthritis and its main types show that this group of chronic diseases are linked with factors that either increase the vulnerability or increase the loading of the joint, as has been previously suggested [26]. On the one hand, social factors, such as low level of wealth, may have increased joints’ vulnerability to be affected by degenerative and/or inflammatory processes. On the other hand, physical and behavioral factors, such as BMI or doing repetitive movements, may have increased the loading within the joints facilitating the manifestation of joint damage.

Being wealthier was associated with less probability of presenting overall arthritis in this community, similar to what has been reported in a population-based study conducted in Brisbane, Australia [24]. Chankom is a Mexican indigenous community, where the people face health inequities [11]. These inequities impede the delivery of timely and appropriate care for solving initial MSK problems for all community members, increasing vulnerability to develop arthritis. Having less wealth in Chankom could also be associated with inadequate nutritional intake, which may foster the progression of joint degeneration and/or inflammation.

Factors that increase joint loading, such as BMI, were significantly associated with a higher prevalence of overall arthritis in this community. This association only held for the prevalence of lower extremity OA, which has been consistently reported in other epidemiologic studies [33]. A person with a BMI of 29 was 1.5 times more likely to present with lower extremity OA than a person with a BMI of 24.

The lack of a significant association observed between BMI and hand OA prevalence does not support the suggested systemic effects of obesity in OA [34]. Consequently, it is possible that in our analysis BMI acted only as a joint loading factor and not as a systemic factor that increased joint vulnerability through serologic inflammatory markers, as has been proposed in the literature [35].

Results related to cumulative mechanical joint stress, the other joint-load increasing factor addressed in this study, were inconsistent and conflict with what has been reported in the literature. On the one hand, static cumulative joint mechanical stress was associated with a lower prevalence of overall arthritis. This association only held for the prevalence of RA, and persons in the RA group reported doing regular repetitive climbing, standing, kneeling, and sitting-to-standing less often than people without RA (Table 3). A recent cross-sectional study conducted in Colombia found that people with RA usually performed low levels of physical impact work [36]. This implies that our findings could be related to a lower engagement in physically demanding activities by the RA group, which can be considered a case of “inversed causality” [37].

On the other hand, the OA subgroup analyses showed that for men, repetitive hand jolting and sit-to-stand movements approached a significant association with hand OA prevalence. In fact, all men with hand OA reported doing these repetitive activities, supporting the notion that dynamic cumulative mechanical joint stresses were linked to the manifestation of this condition. However, repetitive lifting was significantly associated with a lower prevalence of hand OA and repetitive standing for longer than 30 min was significantly associated with a lower prevalence of low-extremity OA in men; while the latter has been linked with a higher prevalence of hip and knee OA [38].

The inconsistent findings observed in the cumulative joint mechanical stress analyses could be related to the high frequency with which participants reported doing repetitive movements during their main occupation. More than 60 % of the population reported doing ≥2 static and dynamic cumulative repetitive movements (data not shown). This indicates some homogeneity among the occupations performed by these community inhabitants; usually, men do the same type of agricultural work while women do similar housework activities. This homogeneity makes it difficult to explore differences between groups. Consequently, we cannot conclude anything solid about the role that joint mechanical stress has on the manifestation of arthritis in this population.

Interestingly, smoking behavior was not significantly associated with RA prevalence in this study, which contradicts several reports in the literature [25, 27]. It has been suggested that only heavy smoking and therefore the dosage, and not just the presence of smoking, is associated with the “seropositive” type of RA [39]. We could not determine whether serologic markers were present in participants with RA. However, we are sure that none of the participants with confirmed RA in Chankom, where smoking is uncommon, reported this behavior. Considering that the prevalence of RA observed in this community (1 %) aligns with the prevalence reported worldwide [27], we could argue against the existence of a real association between smoking and the manifestation of RA.

### Strengths and limitations

The main strengths of this study are related to the methods used for screening and defining arthritis cases and the use of locally grounded measurement instruments. The census strategy, involving the majority of adults living in Chankom, allowed us to conduct a comprehensive analysis of the OA and RA problems in this community. The COPCORD methodology we followed has been validated and used with success in detecting MSK diseases, including OA and RA at the community level in Mexico [40]. This methodology involved a duplicate assessment of cases, including diagnostic confirmation by specialists, which increases our confidence in the validity of the prevalence estimates observed. Finally, the use of a cross-culturally validated instrument, which involved the participation of local people in its development, increased confidence about the local relevance of observed results. For example, people who lived in Chankom decided the properties on which to differentiate levels of wealth among community members, increasing the cultural relevance of the measurements “level of wealth.”

The main limitations of this work are related to the cross-sectional design, the measurement of disability, and the measurement of regular repetitive movements performed in the main occupation. The cross-sectional nature of this study precludes us from establishing causal associations between known risk factors and arthritis incidence. For instance, the accuracy of the counterintuitive associations observed between mechanical joint stress and a lower prevalence of hand and low extremity OA in men can only be established through longitudinal data. The cross-sectional design also prevents the further assessment of “non-specific arthritis” cases, limiting the possibility of observing how these cases progress over time and with which type of arthritis (inflammatory or degenerative) they will ultimately be diagnosed. Responding to this design-related limitation, we initiated a longitudinal surveillance of this population and results will be available in the future.

The measurement of disability is complex and it has been suggested that considering only one dimension of physical function, such as what people think they can do from a pre-defined list of activities contained in a questionnaire is not enough to understand the whole disabling effects of an illness [41]. Therefore, we may not have detected the entire disabling effects of arthritis for people living in Chankom. This is a common limitation in epidemiologic studies of arthritis-related disability. Finally, we did not incorporate a measurement of the actual time (hours, days, months or years) spent on doing the repetitive movements explored in the main occupation. The lack of this temporal component limits our analysis and interpretations about the role that cumulative joint mechanical stress plays in the presentation of arthritis in this community.

### Implications for practice and policy

Overall, arthritis produces high rates of disability in this indigenous population. From a “social determinants of health” perspective, it seems that the conditions of social disadvantage faced by this rural community result in health inequities that condition the manifestation of arthritis. This social disparity has also been observed in a large Mexican multilevel epidemiological study where the prevalence of OA was clearly associated with higher social underdevelopment [42] and in a large Latin American study where the rheumatoid arthritis disabling effects were higher in people of low socioeconomic status [43].

Consequently, there is a need to develop a culturally appropriate community-based rehabilitation intervention directed to prevent the manifestation of arthritis and decrease the associated disabling effects. This intervention should include the early detection and management of RA, as this will potentially improve the functioning outcomes for this population. Appropriate local and regional health policy analyses and strategies need to be undertaken to increase community access to proper health care for people living with chronic MSK diseases in Chankom and other indigenous rural communities. These strategies should also target a reduction of BMI of the adult population as a way to decrease the prevalence of OA in these communities. Finally, our findings support the call for implementing rheumatologic disease prevention and control strategies in Latin American countries [44].

### Implications for research

The disabling effects of arthritis need to be further assessed by incorporating measurements of other dimensions of physical function such as the execution of standardized tasks or the limitations conditioned by the disease on the performance of real life activities. In addition, it is important to evaluate the presence of modifiable factors linked with the progression of arthritis in this community and how these relate with its disabling effects. The association between social factors, such as the level of wealth, and the prevalence of arthritis in Mayan rural communities, should be further explored using quantitative and qualitative methods. Finally, there is a need for longitudinal studies that explore possible causal associations between the significant factors detected in this study and the prevalence of the various arthritis types, especially for those unexpected and counterintuitive associations (i.e., cumulative mechanical joint stress analyses).

## Conclusions

Overall, arthritis is a common chronic condition in Chankom and an important source of disability. Higher level of wealth was associated with lower arthritis prevalence, while higher body mass index was associated with higher OA prevalence. Action is required to decrease the prevalence and disabling effects of these chronic diseases in this community.
